# Detection of New Translocation in Infant Twins with Concordant ALL and Discordant Outcome

**DOI:** 10.3390/pediatric13010002

**Published:** 2020-12-24

**Authors:** Golamreza Bahoush, Maryam Vafapour, Roxana Kariminejad

**Affiliations:** 1 Ali-Asghar Children Hospital, Faculty of Medicine, Iran University of Medical Sciences, Tehran, Iran; vafapourm@gmail.com; 2 Cytogenetic Division, Kariminejad-Najmabadi Pathology & Genetics Center, Tehran, Iran; Roxana_kariminejad@yahoo.com

**Keywords:** acute lymphoblastic leukemia (ALL), monozygotic twins, translocation, MLL rearrangement

## Abstract

About 2–5% of acute lymphoblastic leukemia (ALL) cases in pediatric patients are infants with an unfavorable prognosis because of high relapse probability. Early detection of the disease is, therefore, very important. Despite the fact that leukemia in twins occurs rarely, more attention has been paid to it in genetic studies. In the present study, through cytogenetic testing, a special case of concordant ALL in monozygotic twins was presented with different outcomes. In spite of an acceptable initial consequence to medical treatment in twins, in another brother (Twin B), early relapse was observed. In the cytogenetic study, both twins expressed *t (4; 11) (q21; q23)* while twin A expressed *t (2; 7) (p10; q10).* No cases have previously reported this mutation. Whether this translocation has a protective role for leukemia with mixed-lineage leukemia (MLL) gene rearrangement is still unclear. The difference in the translocation identified in the identical twins is also subject to further investigations.

## 1. Introduction

One of the most common malignancies that could happen between the ages of 2–6 is ALL. Those under the age of 12 months have an extremely poor prognosis [1,2,3,4]. Patients who go into remission have a high incidence of early relapse [1].

In monozygotic twins, the high rate of concordance of leukemia, early onset of infant ALL, and observing similar genetic alterations all suggest that infantile leukemia has a single uterine clonal origin and intra-placental metastasis. *MLL-AF4* fusion sequences have been detected in the Guthrie cards from patients with infantile leukemia [5]. The mixed-lineage leukemia (MLL) gene that is known as histone-lysine N-methyltransferase 2A (*KMT2A* Protein) and is located on chromosome *band 11q23*, was observed approximately in about 34–50% of infant acute myeloid leukemia (AML) cases and 50–80% of infant ALL cases [6]. In older children, the *MLL* gene rearrangements percentage is 14% and 6% for AML and ALL cases, respectively [7]. Infants who are negative for MLL rearrangements have superior outcomes [8]. Based on the studies carried out for investigating concordant leukemia in monozygotic twins have demonstrated that their cancerous cells of leukemia share similar clonotypic markers that indicate an origin in one twin in the womb [9,10]. Additionally, chromosomal translocations are primarily responsible for leukemogenesis events during the embryonic period that indicates MLL gene fusions in infants with ALL [5,11,12].

## 2. Case Presentation

**Figure 1:**
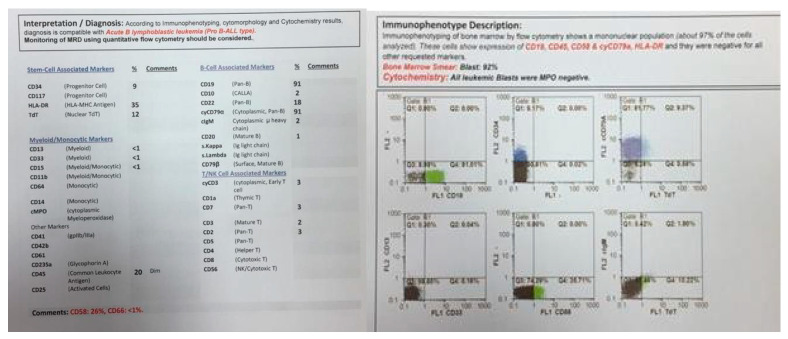
Flowcytometry of twin A.

**Figure 2:**
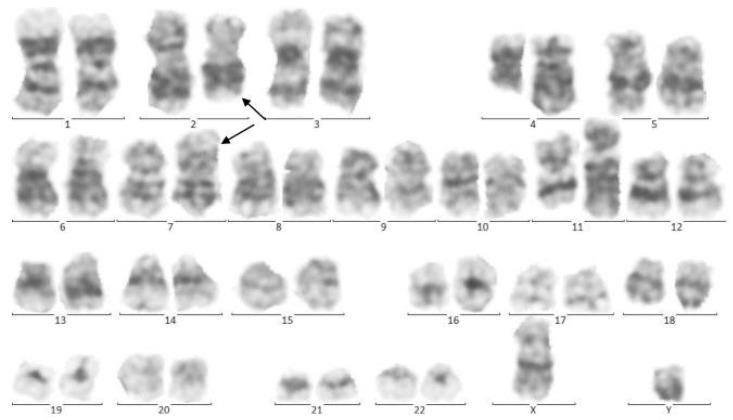
Cytogenetic analysis of twin A. The Metafer-based metaphase finder (MSearch) was used for scanning the slides in this study. Twenty metaphase spreads were selected randomly and studied based on the technique of Greasing the groove (GtG) at 350–400 band resolution. This indicates 46 chromosomes with chromosomal translocation of 4 and 11 chromosomes, which break in 12 spreads where reunion and breakage have occurred at bands 11q23 and 4q21. In four spreads, chromosomes 2 and 7 translocations with breakage and reunion among bands 7p21 and 2p22 was also observed. The remaining eight spreads revealed a normal 46, XY pattern. Conclusion: *46,XY,t(4;11)(q21;q23)/46,XY,idem,t(2;7)(p10;q10)/46, XY*.

**Figure 3:**
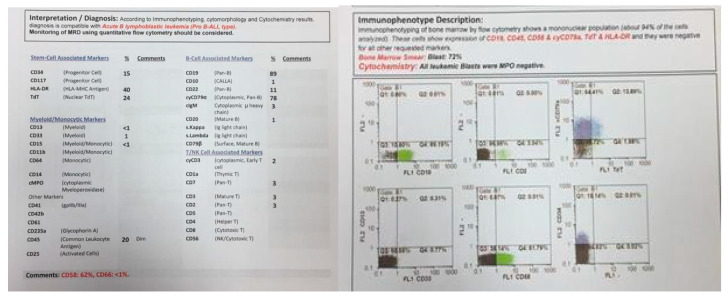
Flowcytometry of twin B.

In 2015, identical twin brothers were born through cesarean delivery at 38 weeks of pregnancy. After examining Twin A for assessment and the ecchymosis history at the age of eight months, it was observed that he had hepatosplenomegaly. The findings of complete blood count (CBC) were as follows: WBC: 85 × 103/μL with many lymphoblasts, Hb: 6.5 g/dl, Plt: 43 × 103/μL. As could be seen from Figure 1, the bone marrow aspiration procedure demonstrated 92% of blasts. Moreover, conducting the immunophenotyping technique of abnormal cells provided the approval of pro-B-ALL diagnosis that is CD10-negative in ALL cases without cytoplasmic immunoglobulin (CIg) or surface immunoglobulin (sIg). Cytogenetic analysis was positive for *t (4; 11*) and *KMT2A-AFF1 (MLL-AF4)* was identified by nested PCR. In accordance with Figure 2, the result of analysis of human G-banded Karyotype in this study was *46, XY, t (4; 11) (q21; q23)/46 XY* idem, *t (2; 7) (p10; q10)/46 XY* (Figure 3). The cerebrospinal fluid was normal.

**Figure 4:**
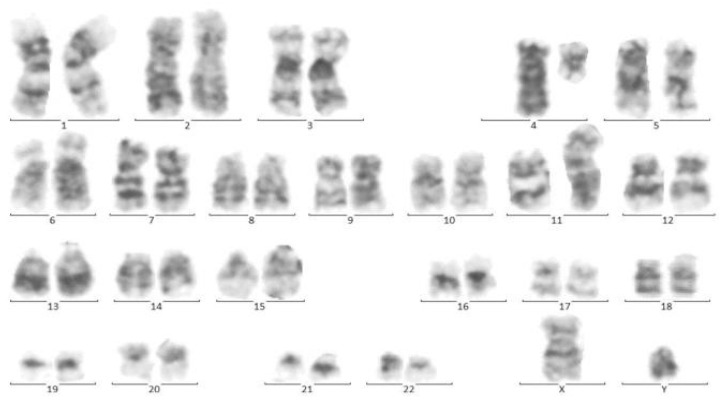
Cytogenetic analysis of twin B. Sixteen metaphase spreads were selected randomly and studied based on the technique of greasing the groove (GtG) at a 350–400 band resolution. That indicates 46 chromosomes with chromosomal translocation of 4 and 11 chromosomes, which breaks in 11 spreads where reunion and breakage have occurred at bands 11q23 and 4q21. The remaining five spreads revealed a normal 46, XY pattern. Conclusion: *46, XY, t (4; 11) (q21; q23)* [11]/*46, XY*.

At the same time, incidental hepatosplenomegaly was detected in twin B. The laboratory data upon admission showed these results: (WBC: 181 × 103/μL with many lymphoblasts, Hb: 9 g/dL, Plt: 148 × 103/μL), with 72% blasts in the bone marrow aspiration. By conducting the immunophenotyping analysis, pro-B-ALL was similarly identified in Twin B (Figure 3). Cytogenetic analysis was positive for *t (4; 11)* and *KMT2A-AFF1 (MLL-AF4)* was identified by nested PCR. As could be seen from Figure 4, the G-banded karyotype was *t (4; 11) (a21; q23)* [11]/*46XY*. Then, in accordance with the Interfant-99 protocol, chemotherapy was used for treating twins with high-risk ALL. About 33 days after applying chemotherapy in twins, the minimal residual disease (MRD) reached complete remission. Twins achieved complete remission with maintenance chemotherapy after 2 years, but twin B relapsed five months after the ending of the protocol.

## 3. Discussion

In 1882, the first report of similar infant twins with concordant leukemia was described [12]. In 1962, Wollman suggested that the origin of infantile leukemia might start from one twin and be transmitted to the other through conjoined circulation [13]. Clarkson and Boyes [14] accepted and developed the hypothesis of metastasis in the placenta. Since the emergence of that hypothesis, a broad range of monochorionic twins have been reported with infantile leukemia, and the development of in vitro techniques has enabled the molecular characterization of the disease.

Estimation of the concordance rate for childhood leukemia was first reported in 1964. In a study by MacMahon and Levy, it is suggested that the concordance rate for acute leukemia (both ALL and AML) is between 5% and 25% in children at birth [15].

Studies have been demonstrated that, in monozygotic twins with ALL, the concordance rate is high. The mechanism that is introduced in the present study has been examined in different studies. In spite of that, both twins may be presented with clinical symptoms of leukemia. These symptoms could not always lead to a precise diagnosis of disease. In this regard, for making an appropriate decision about any possible risks, at the initial steps of examination, healthy twins must be detected and be followed up.

By identifying similar gene variations from leukemic cells, the process of identification of intrauterine monoclonal origin was carried out between infant twin pairs with *MLL* gene abnormalities [16]. Translocation of the MLL gene in two groups of patients including infants and those pediatric patients who were treated previously with topoisomerase II inhibitors for other cancers [11]. Gu et al. [17] demonstrated that there is a significant association among the *MLL* gene and infantile leukemia. In their study, Rubnitz et al. [18] investigated the presence of *MLL* gene rearrangements in infant patients with ALL whether they have *t (4; 11)* or not and/or other structural chromosomal variations involving *11q23*. They introduced the *MLL-AF4* fusion transcript as the most critical prognostic factor in infant patients with ALL.

So far, the exact number of twins with infant leukemia has not been reported. In 2003, more than 70 infants were diagnosed with concordant infantile leukemia, who were same-sex or known monozygotic twin pairs. Concordant leukemia is rare among twins with different sexes or known dizygotic twin pairs [19].

Previous studies have reported that, in some discordant cases of monozygotic twins detected with leukemia, *MLL* gene translocation could be observed. In one case, the placenta was unknown and, in another one, it was dichorionic. However, in some cases, it was thought that the placenta initiation will happen postnatally [20,21]. In this regard, the need to further studies on discordant leukemia in twins seems urgent.

Among twin cases with ALL, the availability of abnormal changes of the chromosome provides adequate evidence of the clonal evolution [22]. In a study carried out by Stumpel et al. [23], the specific pattern of translocation of methylated DNA was identified among *MLL*-rearranged infant ALL and then the effect of DNA hypermethylation on the survival of patients without recurrence was confirmed. The process of identification of overexpression of B-cell lymphoma 2 as an anti-apoptotic factor was carried out in primary cells and cell lines with translocation of *MLL* with *t (4; 11)* [24]. This has led to development of molecularly directed therapy.

Kadan-Lottick et al. have conducted a study on the twin’s cancer risks. They investigated the cancer family history of 211 twins that participated in the Childhood Cancer Survivor Study (CCSS). They also calculated proband-wise twin concordance rates. The achieved results declared that seven pairs of monozygotic twins were concordant to familial cancer (one of them with non-Hodgkin’s lymphoma (NHL), and six other twins with leukemia). For all cancers, the concordance rate was 9.5% (20.0% for NHL and 20.7% leukemia). However, in dizygotic twins, there was no concordance [25]. As a consequence, through the investigated cases, it was shown that unified cytogenetic characteristics are integrated with monozygotic twins. Anyway, in the twins, exogenous factors may also be involved within the leukemia development after in utero initiation.

Appropriate detection of chromosome translocation and rearranged *MLL* gene are two clinical important factors that should be considered for the prognosis and therapy of ALL. Further studies are required to clarify the mechanism underlying this phenomenon. In the present study, an infant leukemia sample in a twin with the same cytogenetic characteristic was investigated. The results were positive for the *MLL-AF4* fusion protein with *t(4;11)* chromosomal translocation. In spite of early appropriate responses for treatment in both twins, one of the brothers experienced an early relapse (Twin B), but twin A continued his complete remission. Two cases of concordant infantile leukemia with discordant outcomes in twins have been previously reported. The first one was reported by Henning Fedders et al. in 2014. In their study, it was suggested that twin cells, with relapse, might evade immune surveillance as a mechanism of disease persistence [26].

Fedders et al. also reported the second case in 2015. Based on their studies, the availability of high levels of DSC2 expression in residual tumor cells may be a pathogenic mechanism of the disease in infantile relapsed ALL patients [27].

Finally, we reported third twins with concordant ALL and discordant outcomes. In the cytogenetic study, twin A expressed *t (2; 7) (p10; q10)*, which was negative in the other twin. No cases have been reported previously for this mutation. Whether this translocation has a protective role for leukemia with MLL gene rearrangement still remains unclear. The difference in the translocation identified in the identical twins is also subject to further deliberation. Evaluation of more twins with infantile leukemia may contribute toward identifying new cytogenetic changes.

## Data Availability

The data that support the ﬁndings of this study are available on request from the corresponding author.
